# Chemogenetic Enhancement of Axon Regeneration Following Peripheral Nerve Injury in the SLICK-A Mouse

**DOI:** 10.3390/brainsci8050093

**Published:** 2018-05-22

**Authors:** Poonam B. Jaiswal, Olivia C. Mistretta, Patricia J. Ward, Arthur W. English

**Affiliations:** Department of Cell Biology, Emory University School of Medicine, Atlanta, GA 30322, USA; pbjaiswal@gmail.com (P.B.J.); olivia.christine.mistretta@emory.edu (O.C.M.); jill.ward@emory.edu (P.J.W.)

**Keywords:** DREADDs, peripheral nerve injury, axon regeneration, CNO, neuroanatomy

## Abstract

The effects of chemogenetics on axon regeneration following peripheral nerve transection and repair were studied in mice expressing a Cre-dependent excitatory designer receptor exclusively activated by designer drugs (DREADD) and Cre-recombinase/yellow fluorescent protein (YFP) in a subset of motor and sensory neurons and cortical motoneurons (SLICK-A). Sciatic nerves were cut and repaired and mice were treated either once, at the time of injury, or five days per week for two weeks with clozapine N-oxide (CNO) (1 mg/kg, i.p.), or were untreated controls. Two weeks after injury, the lengths of YFP+ axon profiles were measured in nerves harvested from euthanized animals. Compared to untreated controls, regenerating axon lengths were not significantly longer in mice treated only once with CNO, but they were more than three times longer in mice receiving CNO repeatedly. Based on results of retrograde labeling experiments, axons of more sensory and motor neurons had regenerated successfully in mice receiving multiple CNO treatments than animals receiving only one treatment or no treatments. The increase in numbers of labeled sensory, but not motor neurons could be accounted for by increases in the proportion of retrogradely labeled neurons also expressing the DREADD. Chemogenetic increases in neuronal excitability represent a potent and innovative treatment to promote peripheral nerve regeneration.

## 1. Introduction

Injuries to peripheral nerves are common. Nearly 200,000 new traumatic peripheral nerve injuries occur in the US each year [1,2]. Although axons in peripheral nerves can regenerate and reinnervate targets in muscle and skin, the process is slow and inefficient, and not all injured neurons participate, leading to poor functional recovery [1]. Successful efforts to enhance the process of axon regeneration have included experimental therapies designed to increase the activity of the neurons whose axons have been injured. These have included brief electrical stimulation and treadmill exercise (reviewed in [3]). Indeed, we have shown, using optogenetics, that increased neuronal activity is both necessary and sufficient for these treatments to promote axon regeneration after peripheral nerve injury [4,5,6].

Chemogenetics has emerged as a means of effecting neuronal excitability, and potentially activity, using a pharmacologic approach. The basis of chemogenetics is designer receptors exclusively activated by designer drugs (DREADDs) [7]. These are muscarinic acetylcholine receptors that have been mutated such that they no longer bind acetylcholine, but display a high affinity for a small molecule designer drug, clozapine-N-oxide (CNO) [8]. Binding of CNO, or its major active metabolite, n-desmethylclozapine [9,10,11], to an excitatory (hM3Dq) DREADD results in activation of a Gq pathway [12] and neuronal excitation [13]. In an earlier report [14], we showed that significant enhancement of axon regeneration was found with CNO treatment in rats induced to express hM3Dq in motoneurons by retrograde adeno-associated viral transduction. Because the CNO treatments did not increase spontaneous electromyographic (EMG) activity, we concluded that DREADD activation must have produced a sub-threshold excitation in the transduced motoneurons, and that this level of excitation was sufficient to promote axon regeneration.

In this study, we evaluated the effects of excitatory DREADD activation in mice in which hM3Dq was expressed genetically in both sensory and motor neurons. We bred mice that express an excitatory DREADD (hM3Dq) in a Cre-dependent manner with a double transgenic mouse known as SLICK (Single neuron labeling with inducible Cre-mediated knockout) [15]. Several strains of SLICK mice have been generated [15]. In all of these mice, yellow fluorescent protein (YFP) and tamoxifen-inducible Cre recombinase are expressed under the control of the thy-1.2 promoter to restrict expression to neurons. In some SLICK strains, YFP and tamoxifen-inducible Cre are widely expressed, but only in neurons. In the strain A (SLICK-A) mice used in this study, YFP is expressed constitutively and Cre recombinase expression is induced after treatment with tamoxifen, but only in a subset of motoneurons and dorsal root ganglion (DRG) neurons with axons in peripheral nerves [15]. It is not known why only a subset of neurons express the transgenes in SLICK-A mice, but this restricted expression enabled us to evaluate axon regeneration in injured nerves directly, by confocal microscopy, and to identify retrogradely labeled neurons expressing DREADDs from those that did not. It is also notable that the YFP and Cre transgenes are also expressed in several other brain regions in these mice, including cortical layer V [16] and in axons in the corticospinal tract [17]. Using neuroanatomical outcome measures, we show here that repeated treatments with CNO in these SLICK-Gq mice resulted in enhanced regeneration of both motor and sensory axons [15].

## 2. Materials and Methods

### 2.1. Animals

Mice expressing a Cre-dependent Gq DREADD (Jackson Laboratories, CAG-LSL-Gq-DREADD; Stock No: 026220) [18] were bred with SLICK-A mice expressing a tamoxifen-inducible Cre recombinase (Jackson Laboratories, B6.Cg-Tg(CAG-cre/Esr1*)5Amc/J; stock no. 004682). Some of the offspring in the F1 generation of this cross expressed both Cre-YFP and hM3Dq, and these animals were used as experimental subjects. Other F1 generation mice expressed neither transgene and these mice were used as graft donors in nerve repair experiments. All animals used in this study were genotyped by Transnetyx Corp. before being included.

To induce Cre recombinase expression, 6–8 weeks old animals expressing both the SLICK-A and hM3Dq genotypes (referred to here as SLICK-Gq) were treated with tamoxifen (Sigma-Aldrich, St. Louis, MO, USA), using a protocol that we have described in detail previously [19]. Briefly, Cre recombinase was activated by two bouts of three consecutive days of oral treatment with tamoxifen, separated by two weeks. Once Cre is expressed, the stop cassette upstream from the DREADD transgene is excised and permanent expression of the Cre-dependent hM3Dq gene occurs. Mice were used in experiments four weeks after the end of the second round of tamoxifen treatments.

All surgical procedures conducted using animals were approved by the Institutional Animal Care and Use Committee of Emory University (Protocol No. 2003261) and conformed to the Guidelines for the Use of Animals in Research of the Society for Neuroscience. All surgical procedures were performed under aseptic conditions. Numbers of mice in each group were chosen based on an a priori power sample size estimate, using a power of 0.8, α = 0.05, and the inter-animal variability observed in our previous studies [20,21].

### 2.2. Nerve Graft Experiments 

For experiments using axon profile lengths as an outcome measure, we used a nerve grafting method we developed several years ago [22]. In Tamoxifen-treated SLICK-Gq mice anesthetized with Ketamine (80 mg/kg; Ketamine HCl, McKesson, Memphis, TN, USA) and Xylazine (10 mg/kg; AnaSed Injection, LLOYD Laboratories, Quezon City, Philippines) the sciatic nerve was exposed bilaterally and cut, using sharp scissors, just proximal to its branching into tibial, common fibular, and sural nerves. The proximal segment of the cut nerve was draped onto a small rectangle of SILASTIC film (Dow Corning 501–1, Thermo Fisher, Norcross, GA, USA). In a similarly anesthetized litter mate to the experimental mouse that expressed neither YFP nor hM3Dq, the sciatic nerve was exposed and a segment approximately 15 mm long proximal to the trifurcation was harvested. The proximal end of this graft was aligned with the proximal segment of the cut nerve in the SLICK-Gq mouse, on the SILASTIC film, and then secured in place using 5–7 µL of fibrin glue: a mixture of fibrinogen, fibronectin, and thrombin (Sigma-Aldrich, St. Louis, MO, USA) [23,24]. The graft was routed beneath the crural fascia and not connected to the distal segment of the cut nerve. Surgical wounds were closed in layers. Post-surgical analgesia was not administered.

Operated mice were studied in five treatment groups (*N* = 5 each). In two control groups, nerves were cut and repaired but were untreated. One group contained tamoxifen-treated SLICK-Gq mice and the other SLICK-A mice that did not contain the hM3Dq transgene. A third group of tamoxifen-treated SLICK-Gq mice received a single treatment with the designer drug, clozapine N-oxide (CNO) (1 mg/kg, i.p.) applied once, immediately after nerve repair had been completed. The fourth group of tamoxifen-treated SLICK-Gq mice received the same dose of CNO for a total of ten times over two weeks. Treatment was begun immediately following nerve repair surgery and continued five days per week for two weeks. The two week time course of this protocol was chosen to be similar that of the exercise protocols that we have used previously to enhance axon regeneration after nerve injury [25]. All CNO treatments were administered in the mornings. In a final group, nerves were cut and repaired in SLICK-A mice that did not express hM3Dq. These animals were exercised, five days per week for two weeks, as we have described previously [19,24]. Animals in this series were euthanized two weeks after nerve repair surgery with an overdose of pentobarbital and perfused transcardially with 0.9% saline and 4% periodate-lysate-paraformaldehyde fixative [19]. Repaired nerves were harvested, cleaned of excess connective tissues, mounted onto microscope slides, and cover-slipped with Vectashield (Vector Laboratories, Burlingame, CA, USA).

**Figure 1:**
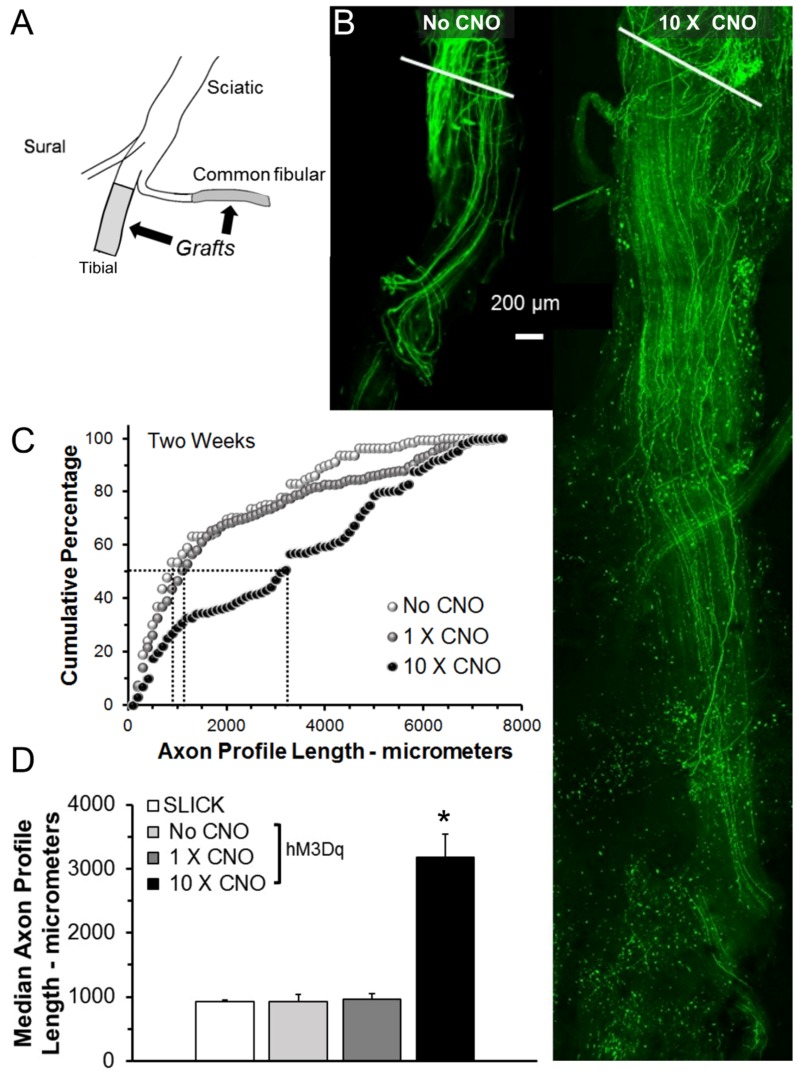
(**A**) Diagram of the experimental approach. (**B**) Images from a single 10-micrometer-thick optical section through repaired nerves from SLICK::hM3Dq mice harvested two weeks after injury. White lines indicate site of sciatic nerve Tx-repair. (**C**) The distributions of axon profile lengths measured two weeks after nerve injury. Dashed lines = medians (**D**) Average median axon profile lengths (±SEM, *N* = 5), * = *p* < 0.05 vs. all others. CNO: clozapine-N-oxide.

Optical sections were made at 10 micrometer thickness through the entire extent of the harvested nerves using a confocal microscope [19], and then stitched together to reconstruct the nerve and graft in three dimensions using software associated with the microscope (Multiphoton Leica SP8, Leica Application Suite-Advanced Fluorescence Software, 3.0.1, Leica Microsystems Inc., Buffalo Grove, IL, USA). Regenerating YFP+ axons are clearly visible in these reconstructions against the dark background of the grafts (see Figure 1B), and all were measured from the surgical repair site to their distal tips, using FIJI (FIJI is just Imagej) software (ImageJ.net). The lengths of regenerating axons were sorted into bins of increasing lengths. The cumulative percentage of total number of these regenerating axons was then calculated. Two analyses were performed on these measurements. First, we evaluated the effects of DREADD activation on axon profile lengths. The distributions of lengths of regenerating YFP+ axon profiles were compared between pairs of groups using the Mann-Whitney U-test. This non-parametric test is used to compare differences between two independent groups when the dependent variable is thought to be not normally distributed. We also evaluated the significance of differences in median axon profile lengths between groups using analysis of variance (ANOVA). Because the distributions of these medians are normal, this parametric analysis was pursued. If the omnibus test returned from the ANOVA was significant (*p* < 0.05), post-hoc paired comparisons (Tukey-Kramer) were performed.

In addition, we evaluated the extent of branching of regenerating axons in the different groups by comparing the numbers of regenerating axon profiles encountered. In SLICK-A mice, we have shown previously [19] that a consistent number of regenerating axon profiles is found in untreated animals two weeks after nerve transection and repair. It is possible that Gq DREADD activation could cause more branching of regenerating axons, as has been observed with some exercise protocols [26]. Thus we evaluated the significance of differences in the numbers of regenerating axon profiles studied using ANOVA and post-hoc testing, where appropriate.

### 2.3. Retrograde Labeling Experiments 

To determine the number of neurons whose axons had regenerated successfully and re-innervated the gastrocnemius muscle, retrograde labeling experiments were performed four weeks after nerve transection and repair. Mice (*N* = 20) were anesthetized and the sciatic nerve exposed, as described above. The intact nerves were secured on a small rectangle of SILASTIC film using fibrin glue. Once secured to the film, the nerve was cut with sharp scissors near the center of the film. This attachment of nerves to the film prevented the withdrawal of its cut segments and helped to align them. A second application of fibrin glue was then applied to secure the cut nerve segments. Deep tissues were then approximated with absorbable 5-0 sutures, and the skin was closed with non-absorbable 5-0 suture. Mice were studied in five treatment groups: untreated SLICK-A mice not expressing hM3Dq, tamoxifen-treated SLICK-Gq mice treated once with CNO, tamoxifen-treated SLICK-Gq mice treated ten times with CNO, and SLICK-A mice not expressing hM3Dq treated either once or ten times with CNO. Four nerve injuries were studied in each group.

Four weeks after nerve repair, the medial and lateral gastrocnemius muscles were exposed in anesthetized mice, and 0.5 µL of a solution of Cholera Toxin Subunit B conjugated to Alexafluor 555 (CTB 555) (Life Technologies, Grand Island, NY, USA, catalog number C-34776) (1 mg/mL in normal saline) was injected into two sites within the lateral gastrocnemius (GAST) muscle using a 30 g needle attached to a Hamilton microliter syringe. Each muscle thus received a total 1 µL of CTB 555 solution. After each injection, the needle remained in place for 30 s prior to withdrawal, and sterile gauze was applied to the needle site for 30 s to minimize potential tracer leakage. The surgical site was washed three times with sterile saline solution. The wounds were closed, and the animals were returned to their cages. All nerve repair surgeries and tracing experiments were performed bilaterally.

After 3–4 days, to allow for retrograde transport of the tracer, animals were euthanized and perfused, as described above. The lumbar spinal cords and fourth lumbar dorsal root ganglia (DRGs) were harvested and cryoprotected. Serial 20 micrometer thick cryostat sections were mounted onto subbed slides and cover-slipped with Vectashield. For counting labeled motoneurons, images of each section were obtained using filter settings appropriate for YFP and Alexafluor 555 and saved to disc. Motoneurons were identified as retrogradely-labeled if the red (CTB 555) label filled the cell soma (with a clear region of the nucleus present) and extended into its proximal dendrites. Care was taken to identify any fragments of each labeled neuron on adjacent serial sections to avoid double counting. Identified neurons that also contained YFP were scored as double-labeled. Mean numbers of tracer-labeled and double-labeled neurons were compared between the five treatment groups using ANOVA, with post hoc testing, where appropriate.

For analysis of labeled DRG neurons, we studied every third serial section from the fourth lumbar ganglion. Analysis was restricted to every third serial section to avoid spurious double counting of small ganglion cells. Cells were identified as containing tracer only or tracer plus YFP, as above. Counts of labeled DRG neurons were compared between the same five treatment groups, as described above.

## 3. Results

### 3.1. Effects of DREADD Activation on Lengths of Regenerating Axons 

In tamoxifen-treated SLICK-Gq mice, peripheral nerves were cut and repaired with grafts harvested from non-fluorescent wild-type littermates (Figure 1A). They were then treated with CNO (1 mg/kg, i.p.), administered either once, immediately following the nerve repair (1 × CNO), or once daily, five days per week for two weeks following nerve repair (10 × CNO). Control tamoxifen-treated SLICK-Gq mice did not receive CNO (No CNO). Two weeks later, the repaired nerves and grafts were harvested from euthanized mice and imaged as whole mounts using confocal microscopy. The expression of YFP in only a subset of the axons in these mice enabled us to visualize and measure regenerating axons over their entire lengths in confocal images. Examples of fluorescent images of grafts reconstructed from optical sections from a control mouse and from a mouse treated 10 times with CNO are shown in Figure 1B. The distributions of axon profile lengths measured in these three groups of mice (*N* = 5 for each group) are shown as cumulative histograms in Figure 1C. The horizontal and vertical dashed lines are used to mark the median (50th percentile) for each of the groups. Note that the distribution for the group treated daily for two weeks (10 × CNO) is shifted significantly (U-test, *p* < 0.01) to the right of the controls (No CNO) as well as that from mice treated only once (1 × CNO). No significant shift was found from controls in the 1 × CNO group. 

Average (±SEM, *N* = 5) median axon profile lengths from these mice are shown in Figure 1D. Results of the omnibus test of a one-way ANOVA for these data were significant (F_3,16_ = 32.60, *p* < 0.01). Based on post-hoc (Tukey-Kramer HSD) analysis, a single treatment with CNO did not result in significantly increased median axon profile lengths relative to either of the control groups. However, treatments with CNO ten times over the two-week period resulted in a greater than three-fold increase in median axon profile lengths, relative to controls. Thus multiple CNO treatments of tamoxifen-treated SLICK-Gq mice resulted in a striking enhancement of axon regeneration.

### 3.2. Effects of DREADD Activation on Numbers of Regenerating Axons 

**Figure 2:**
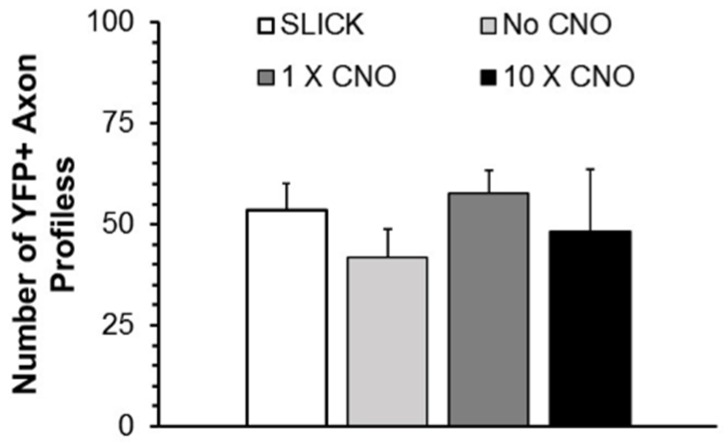
The mean number (±SEM, *N* = 5) of regenerating YPF+ axon profiles found in grafts in the same groups as Figure 1. No Significant differences were found between groups (ANOVA). SLICK: Single neuron labeling with inducible Cre-mediated knockout; CNO: clozapine-N-oxide; YFP: yellow fluorescent protein.

The total number of profiles of YFP+ axons that was measured in each nerve studied was determined and compared between groups to evaluate whether the CNO treatments induced significant axon branching. Mean (±SEM) numbers of YFP+ regenerating axon profiles encountered in mice in the same experimental groups are shown in Figure 2. Using ANOVA, no significant differences were encountered between groups. DREADD activation did not result in a significant change in the number of profiles of regenerating axons.

### 3.3. Effects of DREADD Activation on the Number of Motoneurons Participating in Axon Regeneration 

**Figure 3:**
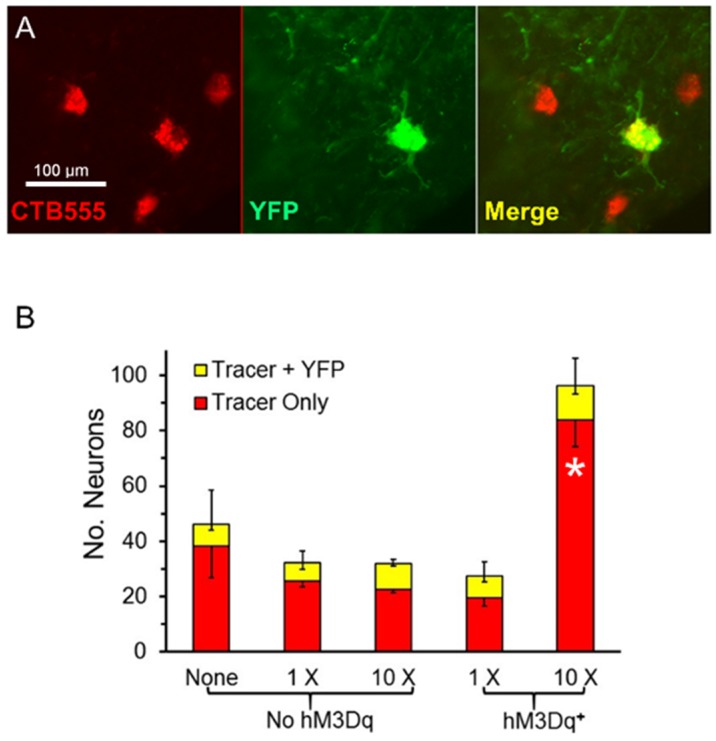
(**A**) Examples of singly and doubly labeled motoneurons in the spinal cord (40×). (**B**) Mean number of retrogradely labeled neurons four weeks after sciatic nerve transection and repair. The red portion of each bar represents the numbers of neurons containing only the retrograde fluorescent tracer used; the yellow portion the number of cells containing both the tracer and YFP. Positive error bars are the SEM (*N* = 4) for total motoneuron (tracer + tracer and YFP) numbers; downward error bars tracer only and tracer + YFP groups. * = *p* < 0.05 vs. all other groups.

The number of motoneurons whose axons had regenerated successfully was determined using retrograde transport of fluorescent tracers injected into the GAST muscles four weeks after sciatic nerve transection and repair. Because the SLICK-A mice used in this study express YFP in only a subset of motoneurons and in all Cre-expressing cells (see above), we were able to differentiate motoneurons containing hM3Dq from those that did not. We counted motoneurons that contained only the retrograde label (Figure 3A, red) and those that contained both the retrograde label and YFP (Figure 3A, yellow). Counts of average numbers (±SEM, *N* = 4 for all groups) of single and double labeled motoneurons are shown in Figure 3B.

The total number of retrogradely labeled motoneurons found four weeks after sciatic nerve transection and repair was more than three times larger in tamoxifen-treated SLICK-Gq mice that received ten treatments with CNO than in any of the other groups. The omnibus test of a one-way ANOVA was significant (F_4,15_ = 16.34, *p* < 0.01). Using post-hoc paired testing, significant (*p* < 0.05) differences were found between the group of mice expressing the Gq DREADD that had been treated ten times with CNO and all other groups, including mice treated with CNO but not expressing hM3Dq. Differences in the mean number of retrogradely labeled motoneurons between the other four groups were not significant. The same was true if the motoneurons marked only by the fluorescent retrograde tracer was considered. The omnibus test of a one-way ANOVA was significant (F_4,15_ = 17.23, *p* < 0.01) and in post-hoc paired testing only paired comparisons involving the mice expressing DREADDs and treated ten times with CNO were significant. However, the number of cells that contained both the retrograde tracer and YFP did not differ significantly among any of the treatment groups (ANOVA, F_4,15_ = 0.92, *p* = 0.48). Thus, repeated treatments with CNO promoted the successful regeneration of axons of motoneurons in tamoxifen-treated SLICK-Gq mice, irrespective of whether those motoneurons expressed the DREADD target of the treatment.

### 3.4. Effects of DREADD Activation on the Number of Dorsal Root Ganglion Neurons Participating in Axon Regeneration 

**Figure 4:**
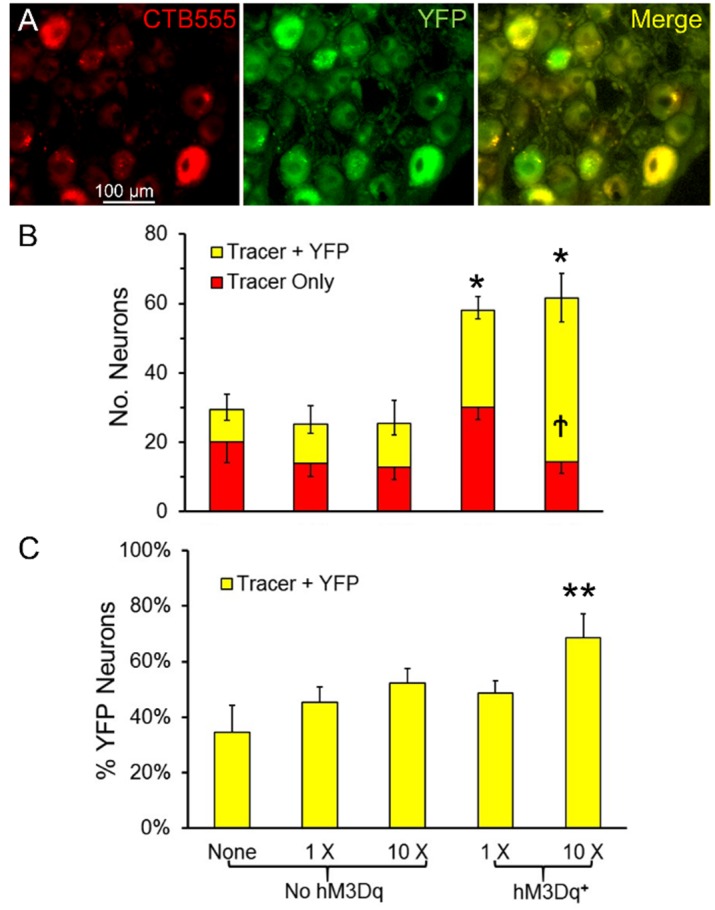
(**A**) Double labeled L4 dorsal root ganglion (DRG) neurons (40×). (**B**) The mean number (±SEM, *N* = 4) of L4 DRG neurons labeled four weeks after nerve injury. The same color coding and error bars are used as in Figure 3. (**C**) The mean proportion of double labeled DRG neurons (±SEM) is shown for the same five groups. * = *p* < 0.05 vs. No hM3Dq for all cells, ** = *p* < 0.05 vs. all other groups, † = *p* < 0.05 vs. all other groups for Tracer + YFP cells.

In the same mice used in the motoneuron experiments described above, labeled L4 DRG neurons were counted and scored as containing the retrograde tracer or the tracer and YFP (double labeled). Sensory neurons containing YFP were assumed to express hM3Dq. Examples of double labeled sensory neurons are shown in Figure 4A. Counts of average numbers (±SEM, *N* = 4 for all groups) of single and double labeled DRG neurons are shown in Figure 4B. The omnibus test from the ANOVA comparing the total number of retrogradely labeled DRG neurons was significant (ANOVA, F_4,15_ = 12.14, *p* < 0.01). Using post-hoc paired testing, significantly more labeled DRG neurons were found in tamoxifen treated SLICK-Gq mice treated with CNO than any of the other three groups, whether the mice were treated once or treated ten times. No significant effect of CNO treatment was found in mice that did not express hM3Dq. Similar numbers of L4 DRG neurons regenerated successfully in these mice as in SLICK-Gq mice not treated with CNO. The proportion of all labeled DRG neurons that were double labeled in mice treated ten times with CNO was significantly (ANOVA, F_4,15_ = 6.25, *p* < 0.01) greater than in all of the other groups (Figure 4C). 

## 4. Discussion

The goal of this study was to evaluate the use of chemogenetics as a means of enhancing sensory and motor axon regeneration after peripheral nerve transection injury using neuroanatomical outcome measures. In mice in which an excitatory DREADD (hM3Dq) and YFP were expressed in a Cre-dependent manner, we evaluated the lengths and numbers of regenerating axons after sciatic nerve injury and the participation and success of regeneration of axons of motor and sensory neurons. The main finding was that repeated Gq DREADD activation by the designer drug, clozapine-N-oxide (CNO) resulted in significant enhancement of axon regeneration. Lengths of profiles of regenerating axons in mice treated ten times over a two week period with CNO were nearly three times longer than controls, or mice treated only once with CNO, and comparable to those we had shown in similar mice using post-injury exercise as a therapy [24]. Axons of more motoneurons and dorsal root ganglion sensory neurons had regenerated successfully and reinnervated a muscle target in the 10 × CNO group than in controls.

The simplest explanation for this chemogenetic enhancement of regeneration is that activation of the Gq DREADD using CNO resulted in an increase in the excitation of the injured neurons, leading to enhanced elongation of regenerating axons—a form of activity-dependent enhancement. In a previous study, we found that background EMG activity was not increased by CNO treatment of rats induced to express hM3Dq in motoneurons [14], leading us to conclude that activation of these modified muscarinic acetylcholine receptors produced an excitation of these neurons that was sub-threshold. Alternatively, constitutive expression of inhibitory DREADDs has been shown to induce CNO independent functional changes in sensory neurons [27]. Whether such changes could be caused by expression of Gq-DREADDs needs to be investigated. We believe that a subthreshold excitation could explain our finding of greater success of sensory axon regeneration found in mice treated daily with CNO. In these mice, axons of significantly more YFP+ L4-DRG neurons was found. Treatments with CNO in SLICK-Gq mice would be expected to increase excitation of those DRG neurons expressing Cre recombinase and hM3Dq (and YFP) and not those that did not express the DREADD. Based on our previous report investigating DREADD activation of motoneurons [14] and a literature report of inhibitory DREADD-based inhibition of DRG neurons [27], we anticipate that the effect of the CNO treatment will be limited to a few hours. Thus, our finding an effect of ten daily treatments, but not a single CNO injection, also might be reasonable.

The enhanced regeneration of motor axons found in the mice treated daily with CNO might not be as easily explained. Although not significantly greater, in these animals, 50% more motoneurons were retrogradely labeled than in any of the other groups studied. We interpret this finding to mean that axons of more motoneurons had regenerated successfully in the mice treated daily with CNO than in the other groups. Thus, the daily CNO treatments enhanced the regeneration of axons substantially more in motoneurons that did not contain YFP, and were thus presumed not to express the hM3Dq, than in motoneurons that did express hM3Dq. One interpretation of this finding might be that the CNO treatments produced an increased excitation of motoneurons irrespective of their Gq DREADD content. Given the well-documented in vivo metabolism of CNO to n-desmethylclozapine [9,10,11], such an interpretation must be considered. To investigate this possibility, one of our control groups that did not express the Cre-dependent hM3Dq transgene was treated daily with CNO. The numbers of retrogradely labeled motoneurons in this group were not significantly different from those of untreated animals or mice treated once with CNO, indicating that the CNO treatments had no effect on regeneration of axons of motoneurons that did not express hM3Dq.

An alternative explanation might be that the daily CNO treatments increased the excitation of other neuronal systems that express both Cre recombinase and hM3Dq, which, could contribute to the excitation of both Gq positive and Gq negative motoneurons. One such system is the corticospinal tract. It is known that in the SLICK-A mice used in this study, neurons in lamina V of the motor cortex and axons in the corticospinal tract express YFP, and tamoxifen-inducible Cre recombinase [17]. Thus, we speculate that multiple systemic treatments of tamoxifen-treated SLICK-Gq mice with CNO could result in corticospinal excitation of the all of the GAST motoneurons, leading to enhancement of regeneration of their cut axons. Continual cortical stimulation has been shown to contribute to significant and long-lasting plasticity in spinal circuitry and enlargement of the monosynaptic H reflex [28]. Whether pharmacogenetic DREADD activation of the corticospinal system could produce similar effects and account for the results presented here awaits further experimentation.

## 5. Conclusions

Chemogenetic activation of excitatory DREADDs in the SLICK-A mouse resulted in enhanced regeneration of both sensory and motor axons following peripheral nerve injury.
